# Dopamine acting at D1-like, D2-like and α1-adrenergic receptors differentially modulates theta and gamma oscillatory activity in primary motor cortex

**DOI:** 10.1371/journal.pone.0181633

**Published:** 2017-07-21

**Authors:** Mazhar Özkan, Nicholas W. Johnson, Umit S. Sehirli, Gavin L. Woodhall, Ian M. Stanford

**Affiliations:** 1 Aston Brain Centre, Aston University, School of Life and Health Sciences, Birmingham, United Kingdom; 2 Department of Anatomy, School of Medicine, Marmara University, Istanbul, Turkey; SUNY Downstate MC, UNITED STATES

## Abstract

The loss of dopamine (DA) in Parkinson’s is accompanied by the emergence of exaggerated theta and beta frequency neuronal oscillatory activity in the primary motor cortex (M1) and basal ganglia. DA replacement therapy or deep brain stimulation reduces the power of these oscillations and this is coincident with an improvement in motor performance implying a causal relationship. Here we provide *in vitro* evidence for the differential modulation of theta and gamma activity in M1 by DA acting at receptors exhibiting conventional and non-conventional DA pharmacology. Recording local field potentials in deep layer V of rat M1, co-application of carbachol (CCh, 5 μM) and kainic acid (KA, 150 nM) elicited simultaneous oscillations at a frequency of 6.49 ± 0.18 Hz (theta, n = 84) and 34.97 ± 0.39 Hz (gamma, n = 84). Bath application of DA resulted in a decrease in gamma power with no change in theta power. However, application of either the D1-like receptor agonist SKF38393 or the D2-like agonist quinpirole increased the power of both theta and gamma suggesting that the DA-mediated inhibition of oscillatory power is by action at other sites other than classical DA receptors. Application of amphetamine, which promotes endogenous amine neurotransmitter release, or the adrenergic α1-selective agonist phenylephrine mimicked the action of DA and reduced gamma power, a result unaffected by prior co-application of D1 and D2 receptor antagonists SCH23390 and sulpiride. Finally, application of the α1-adrenergic receptor antagonist prazosin blocked the action of DA on gamma power suggestive of interaction between α1 and DA receptors. These results show that DA mediates complex actions acting at dopamine D1-like and D2-like receptors, α1 adrenergic receptors and possibly DA/α1 heteromultimeric receptors to differentially modulate theta and gamma activity in M1.

## Introduction

In Parkinson’s, the loss of dopamine (DA) is characterised by the emergence of high-amplitude oscillations that predominate at theta (4–8 Hz) and beta frequency (15–35 Hz) [1,2,3,4]. This increase in beta oscillatory power, in cortex and nuclei of the basal ganglia, is thought to limit computational capacity [5] through reduced information coding and consequent inability to uncouple and re-recruit appropriate motor networks. Administration of levodopa [6,7] or deep brain stimulation of the subthalamic nucleus [8,9] reduces beta power, which is accompanied by improvement in motor performance.

Recent evidence has implicated the primary motor cortex (M1) as a potential target both for pharmacological and surgical interventions in Parkinson’s. Stimulation of M1 appears to normalise firing rate of neurons in the subthalamic nucleus producing immediate improvements in tremor, rigidity, as well as long-term improvements in bradykinesia and akinesia [10,11], while repetitive transcranial magnetic stimulation of M1 improves motor performance in Parkinson’s [12,13,14,15]. In addition, antidromic stimulation of deep layer motor cortical pyramidal cells [16] and recent advances using optogenetic approaches have shown that afferent axons projecting from deep layers of M1 may be the primary target in effective deep brain stimulation [17].

For a number of years we have investigated network oscillatory activity in brain slices recording from M1 [18,19,20,21,22] and observed that oscillatory activity can be routinely induced by co-application of the muscarinic receptor agonist carbachol (CCh) and the glutamate receptor agonist kainic acid (KA). Beta and gamma oscillations in M1 are abolished by picrotoxin and modulated by gabazine, tiagabine and zolpidem, indicating dependence on networks of fast spiking (FS) GABAergic interneurons [18,21,22]. As with gamma (35–80 Hz) oscillations in the hippocampus [23,24] beta oscillatory activity in M1 is generated as a consequence of sustained excitation of networks of inhibitory interneurons [18,20] which are able to entrain each other to fire in a synchronous manner and hence sculpt pyramidal cell activity through repetitive (I_phasic_) inhibitory discharges, the frequency of which is dependent upon the kinetics of the inhibitory postsynaptic potentials [23,25,26].

DA projections preferentially innervate deep layers V and VI of the M1 and prefrontal cortex [27,28,29,30,31] and particularly the parvalbumin-positive FS interneurons therein [32,33]. Furthermore, both D1 and D2 receptors have been observed in deep layers [34] with D1 receptor immunoreactivity being prevalent on parvalbumin-positive interneurons [35,36,37]. Therefore, as FS cells are rhythmogenic governors of projection neurons, it might be expected that DA would have a profound effect on the oscillatory activity in M1.

Using an improved brain slice preparation based on a protocol utilising a number of neuro-protectant agents, we have previously reported simultaneous persistent theta and gamma oscillations in M1, which appear mechanistically distinct [22]. In this study we show differential modulation of theta and gamma oscillations by DA acting at both D1 and D2-like receptors, α1 adrenergic receptors and possibly DA/ α1 heteromultimeric receptor complexes.

## Materials and methods

### In vitro slice preparation

All animal procedures were performed in accordance with the Aston University policy on research involving animals and under a project license approved by the Aston University Bioethics Committee. Procedures were also in accordance with the Animals (Scientific Procedures) Act UK 1986 as amended by the European Communities Directive 2010/63/EU. Sagittal brain slices (450 μm thick) containing M1 were prepared from male Wistar rats (50–100 g). Animals were first anaesthetised using isoflurane (4% in O_2_) until no heartbeat was detected and then transcardially perfused with ice-cold sucrose-based artificial cerebral spinal fluid (aCSF) containing (in mM): 180 sucrose, KCl 2.5, MgSO_4_ 10, NaH_2_PO_4_ 1.25, NaHCO_3_ 25, glucose 10, CaCl_2_ 0.5, ascorbic acid 1, N-acetyl cysteine 2, taurine 1, ethyl pyruvate 20 and saturated with 95% O_2_ and 5% CO_2_ at pH 7.3, 300–310 mOsm. Indomethacin (45 μM), aminoguanidine (200 μM) and uric acid (400 μM) were added to improve slice viability [22]. The brain was quickly removed and placed into the same sucrose-based aCSF. Using a HM-650V Microslicer (Microm GMBH, Germany) sagittal slices were cut at room temperature. Slices were then transferred to an interface holding chamber at room temperature containing oxygenated standard aCSF containing (in mM): NaCl 126, KCl 3, MgSO_4_ 1.6, NaH_2_PO_4_ 1.25, NaHCO_3_ 26, glucose 10, CaCl_2_ 2, with an osmolarity between 300–310 mOsm, where they were left for at least 1 hour.

### Extracellular recordings

Slices were transferred to an interface recording chamber (Scientific System Design Inc., Canada) at 32–34°C and continually perfused at 1–2 ml/min with standard aCSF. Local field potential (LFP) recordings in deep layer V of M1 were made using borosilicate glass microelectrodes pulled on a Flaming-Brown micropipette puller (P-1000; Sutter Instrument Co., USA) filled with standard aCSF (resistance of 1–3 MΩ). Signals were amplified 1000-fold using an EXT10-2F amplifier and an LHBF-48X filter (NPI Electronics GMBH, Germany), band-pass filtered at 0.5 Hz and 700 Hz. Low amplitude 50 Hz signal interference was removed using a HumBug (Quest Scientific, North Vancouver, Canada). Signals were digitized and recorded at 10 kHz using a CED Micro-1401 mkII digitizer and Spike2 software (Cambridge Electronic Design, UK) and saved to disk.

### Drug application

Oscillatory activity was induced by bath application of CCh (5 μM) and KA (150 nM) and left to stabilise for at least 60 min prior to recording. Drugs were bath applied in known concentrations having been previously prepared in stock solutions of 1–50 mM and stored at -20°C. The drugs used were carbamoylcholine chloride (carbachol), DA and amphetamine (Sigma Ltd., Gillingham, UK), kainic acid (Abcam, Cambridge, UK), SKF38393, SCH23390, quinpirole, sulpiride, prazosin, phenylephrine (Tocris Bioscience, Bristol, UK). All drugs were applied for a minimum of 40 min before data were sampled.

DA is readily oxidised (observed with notable colour change) which will alter the effective concentration and potentially produce unreliable results. DA oxidation may also result in the production of reactive oxygen species and free radicals which may have a detrimental effect on slice viability. We found that the addition of the anti-oxidant ascorbic acid [38,39] the aCSF prior to DA application, successfully prevented the oxidation of DA for the duration of our experiments. Baseline recordings for all experiments were therefore recorded in the presence of ascorbic acid (500 μM).

### Data analysis

Data were analysed off-line using Spike2 (CED, UK). Raw data presented were filtered using IIR digital filtering by Bessel band pass between 3–10 Hz for theta oscillations and 30–45 Hz for gamma oscillations. Changes to power values were derived from power spectra generated with Fourier analysis of 40 s epochs of data from control and drug-applied conditions. Unless otherwise stated, pooled data are represented as mean peak power values normalised to control ± SEM. The Wilcoxon signed rank test was used for analysis of paired data sets while the Kruskal-Wallis test followed by Dunn's multiple comparisons test was used for the analysis of experiments using multiple doses of the same drug. All tests were performed using GraphPad Prism version 5.00 for Windows (GraphPad Software, San Diego California USA). Significance expressed as *p<0.05, **p<0.01, ***p<0.001. Significant changes in power are given in Table 1 with changes denoted by arrows (up represents an increase in power and a down arrow, a decrease power. The number of arrows corresponds to significance at 1, 2 or 3*).

**Table 1:** A summary of the drug induced changes in theta and gamma power.

Drug	N (slice)	Theta power	Gamma power
Dopamine (30 μM)	8	**-**	↓
SKF38393 (10 μM)	25	↑↑↑	↑↑↑
Quinpirole (10 μM)	13	↑↑	↑
Amphetamine (20 μM)	13	↑↑↑	↓↓↓
Phenylephrine (10 μM)	9	-	↓↓
Prazosin (10 μM)	10	↑↑	**-**
Dopamine (in SCH23390 + Sulpiride)	6	↑	**-**
Dopamine (in Prazosin)	10	↑↑	**-**
Phenylephrine (in SCH23390 + Sulpiride)	7	↓↓	↓↓

## Results

**Fig 1:**
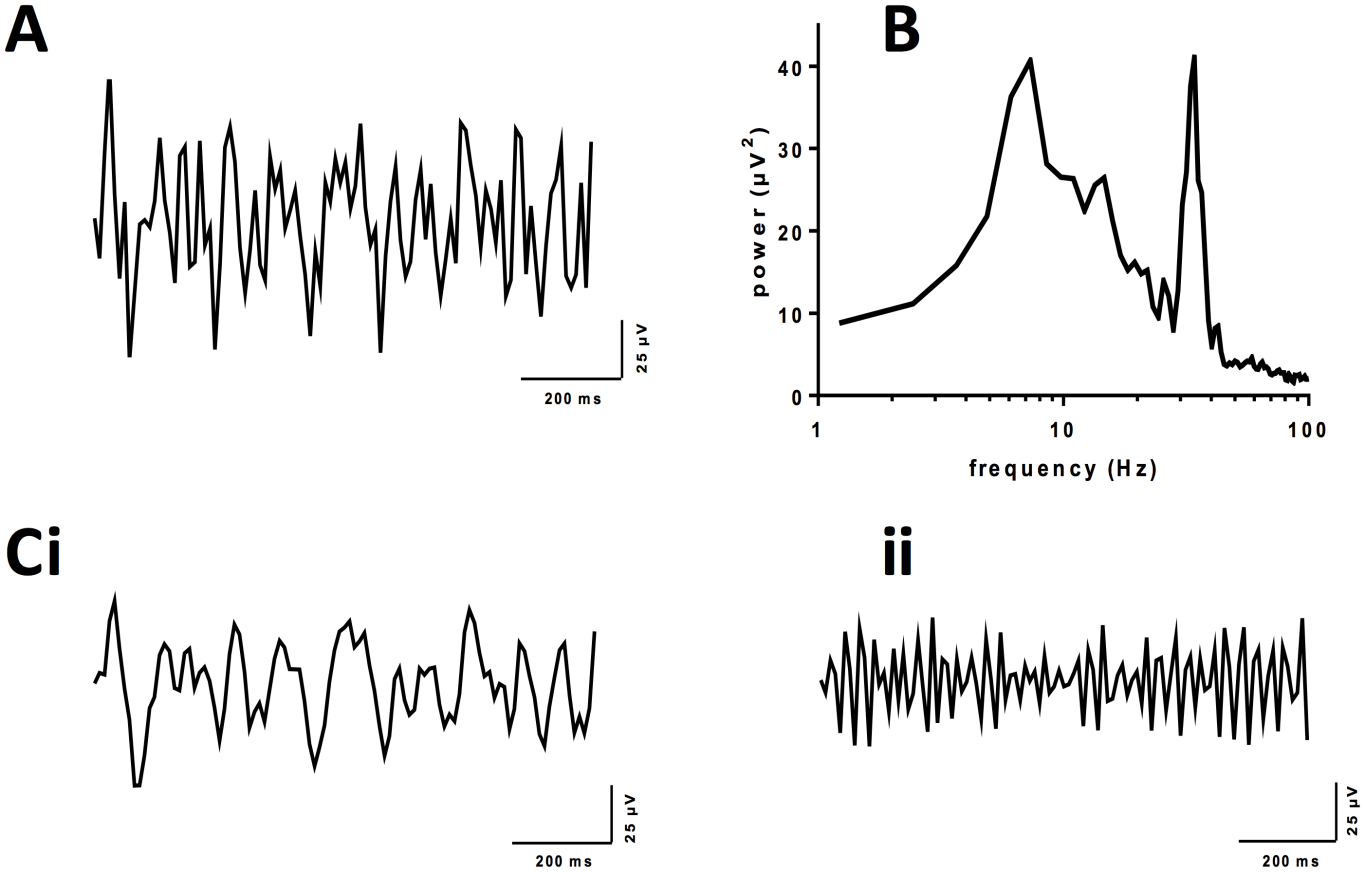
Simultaneous theta and gamma oscillations in M1. (A) Extracellular *in vitro* recording from deep layer V of M1 in the presence of CCh (5 μM) and KA (150 nM) which has been low-pass filtered at 60 Hz. (B) Representative power-spectrum demonstrating emergence of theta and gamma frequency oscillatory activity (C) The same data after band-pass filtering showing (i) theta (3–10 Hz) and (ii) gamma (30–45 Hz) oscillations.

Previously, CCh and KA at 50 μM and 400 nM respectively were used to generate beta oscillations in M1 [18]. Here, we have applied KA and CCh at lower concentrations and observed the emergence of multiple rhythms (Fig 1A and 1B). These differences presumably reflect the addition of neuro-protectants which improved slice viability and enhanced preservation of the GABA interneurons required for rhythmogenesis. Therefore, recording in deep layer V of M1, application of CCh (5 μM) and KA (150 nM) generated simultaneous rhythms at theta frequency (6.49 ± 0.18 Hz, n = 84, Fig 1Ci) and gamma frequency (34.97 ± 0.39 Hz, n = 84, Fig 1Cii).

**Fig 2:**
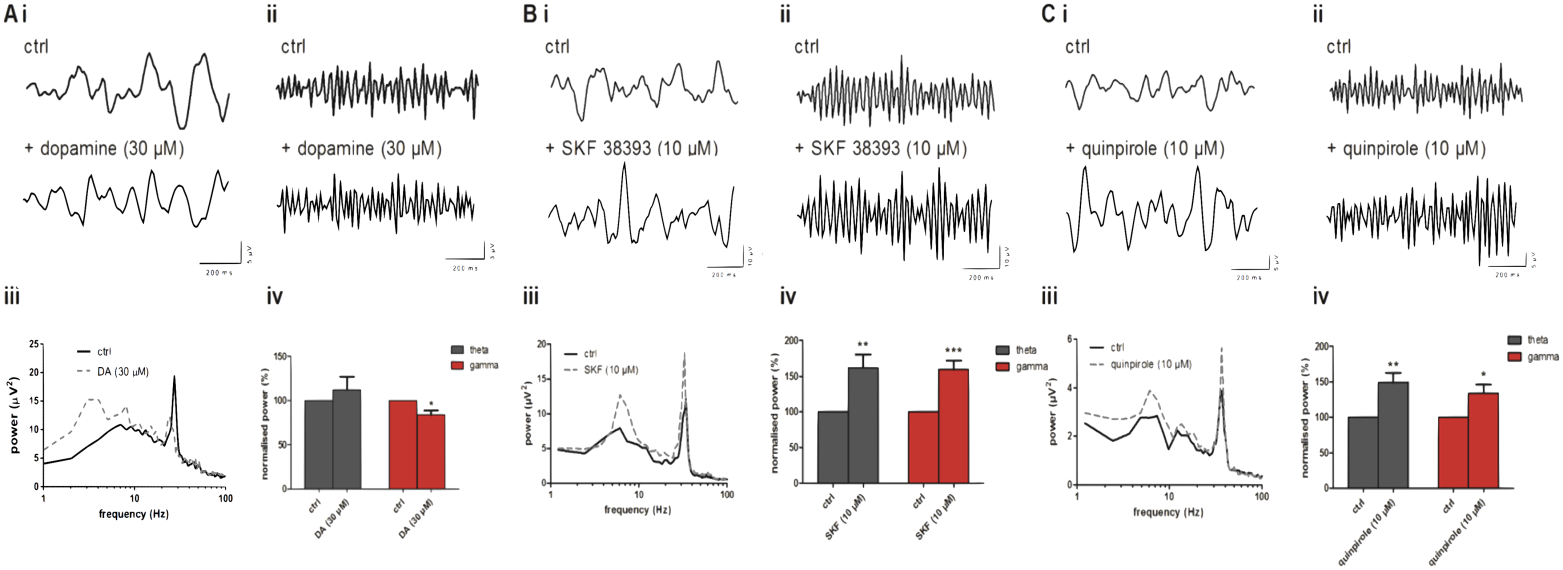
DA reduces gamma power while DA receptor agonists increase both theta and gamma power. (A) Band-passed raw data of (i) theta and (ii) gamma oscillations after induction with CCh and KA (ctrl) and upon application of dopamine (30 μM). (iii) Typical power spectra before (solid line) and after application of dopamine (dashed line). (iv) Peak power changes of theta (grey bars) and gamma (red bars) oscillations normalised to control. (B) Band-passed raw data of (i) theta and (ii) gamma oscillations after induction with CCh and KA (ctrl) and after application of SKF 38393 (SKF, 10 μM). (iii) Typical power spectra demonstrating peak responses before (solid line) and after application of SKF (dashed line). (iv) Peak power changes of theta (grey bars) and gamma (red bars) oscillations normalised to control. (C) Band-passed raw data of (i) theta and (ii) gamma oscillations after induction with CCh and KA (ctrl) and after application of quinpirole (10 μM). (iii) Typical power spectra demonstrating peak responses before (solid line) and after application of quinpirole (dashed line). (iv) Peak power changes of theta (grey bars) and gamma (red bars) oscillations normalised to control. * p<0.05, **p<0.01, ***, p<0.001.

Exogenous application of DA (30 μM) was without effect on theta oscillatory power (112.4 ± 14.6%, n = 8, ns Fig 2Ai, iii, iv) but significantly reduced gamma oscillations (83.9 ± 4.9%, n = 8, p<0.05, Fig 2Aii, iii, iv). In subsequent experiments, we applied the D1-like receptor agonist SKF38393 (SKF; 10 μM) or the D2-like receptor agonist quinpirole (10 μM). Application of SKF resulted in the significant increase in the power of both theta (162 ± 17.9%, n = 25, p<0.001, Fig 2Bi, iii, iv) and gamma (160 ± 12.4%, n = 25, p<0.001, Fig 2Bii, iii, iv) oscillations. Likewise, application of quinpirole significantly increased the power of both theta (to 149.0 ± 13.8%, n = 9, p<0.01, Fig 2Ci, iii, iv) and gamma oscillations (to 133.9 ± 12.3%, n = 9, p<0.05, Fig 2Cii, iii, iv).

**Fig 3:**
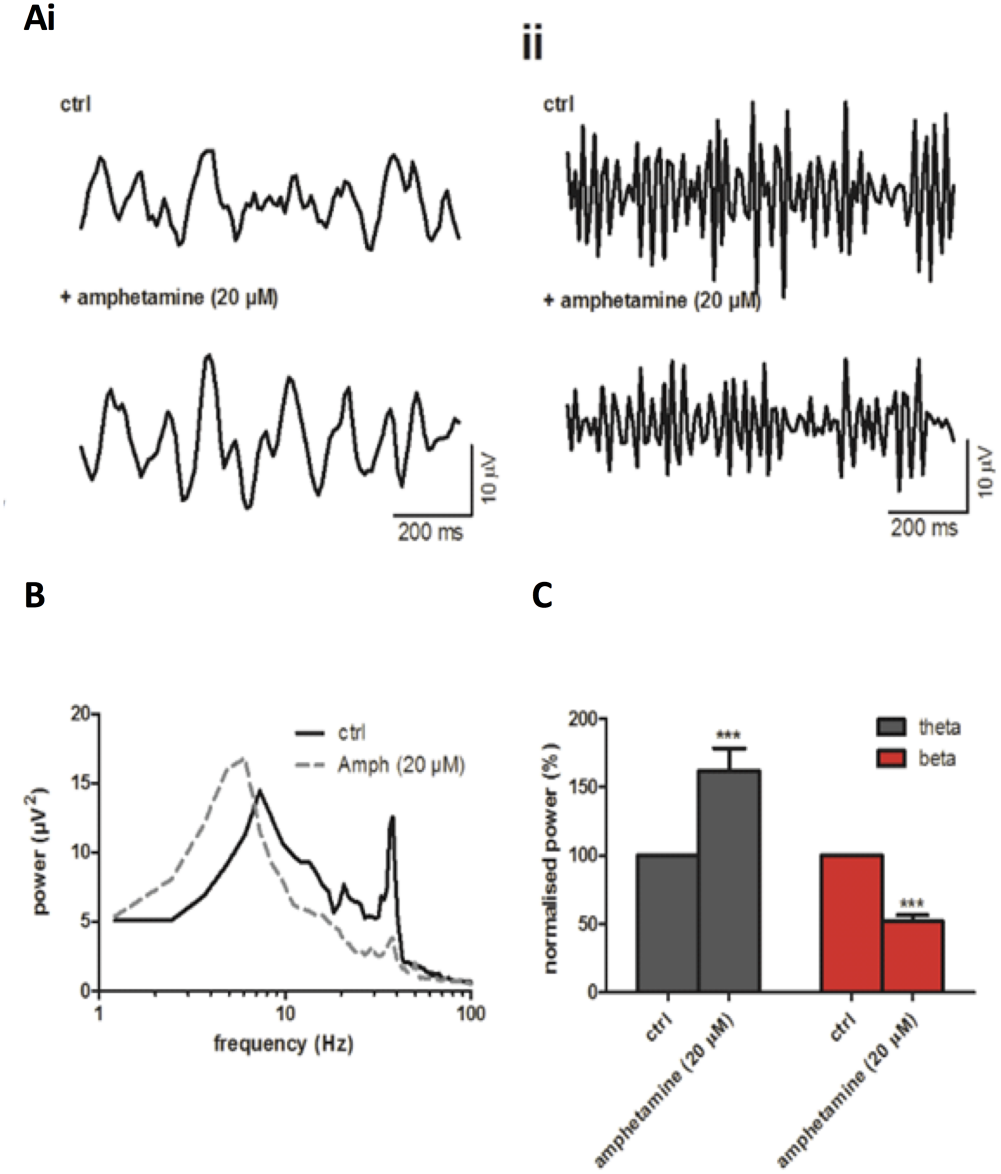
Amphetamine increases theta power while reducing gamma power. (A) Band-passed raw data of (i) theta and (ii) gamma oscillations after induction with CCh and KA (ctrl) and upon application of amphetamine (20 μM). (B) Typical power spectra before (solid line) and after application of amphetamine (dashed line). (C) Peak power changes of theta (grey bars) and gamma (red bars) oscillations normalised to control. ***, p<0.001.

In order to avoid exogenous application of DA which is prone to oxidation even in the presence of ascorbic acid, we applied amphetamine to promote endogenous release of DA [see 40 for review]. However, it should be noted that amphetamine may also release noradrenaline, 5-HT and acetylcholine as well as DA [40,41,42]. Bath application of amphetamine (20 μM) increased theta power (151 ± 16.2%, n = 13, p<0.001 Fig 3Ai, 3B and 3C) and decreased gamma power (67 ± 7.8%, n = 13, p<0.001 Fig 3Aii, 3B and 3C).

**Fig 4:**
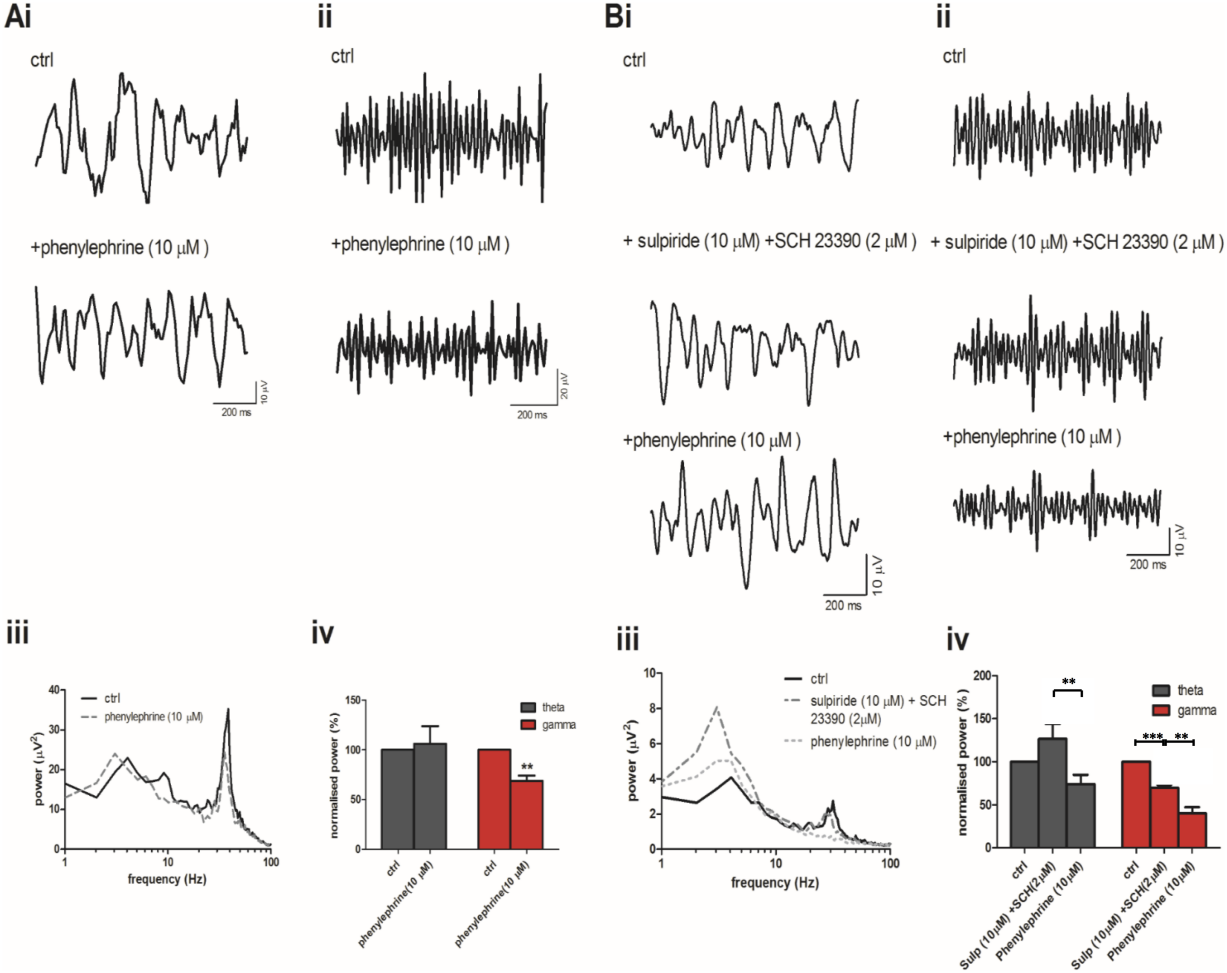
DA action is mimicked by the α1 adrenergic receptor agonist phenylephrine. (A) Band-passed raw data of (i) theta and (ii) gamma oscillations after induction with CCh and KA (ctrl) and after application of phenylephrine (10 μM) (iii) Typical power spectra demonstrating peak responses before (solid line) and after application of phenylephrine (dashed line). (iv) Peak power changes of theta (grey bars) and gamma (red bars) oscillations normalised to control. (B) Band-passed raw data of (i) theta and (ii) gamma oscillations after induction with CCh and KA (ctrl) and after application of sulpiride (10 μM) and SCH (2 μM) and then addition of phenylephrine (10 μM). (iii) Typical power spectra demonstrating peak responses before (solid line) and after application of sulpiride and SCH (dashed line) and then upon addition of phenylephrine (dotted line). (iv) Peak power changes of theta (grey bars) and gamma (red bars) oscillations normalised to control **p<0.01, *** p<0.001.

These data showed that application of DA receptor agonists had different effects to those of exogenous or endogenous DA. As recent evidence has suggested that DA may also act on adrenergic receptors [43,44], we decided to pharmacologically assess the role of α1-adrenergic receptors. Application of the α1-adrenergic receptor agonist phenylephrine (10 μM) had no effect on theta power (106 ± 7.8%, n = 9, ns, Fig 4Ai, iii, iv) but significantly reduced gamma power (69 ± 5.2%, n = 9, p<0.01, Fig 4Aii, iii, iv). Hence, it is possible that DA acts through α1-adrenergic receptors. We repeated the application of phenylephrine in the presence of a combination of D1 receptor antagonist SCH23390 (SCH, 2 μM) and the D2 receptor antagonist sulpiride (10 μM). Under these conditions phenylephrine, induced a significant reduction in theta power (to 74.2 ± 11% of control, p<0.01 vs SCH + sulpiride, n = 7, 4Bi, iii, iv) and a significant reduction in gamma power (to 40.5 ± 7% of control, p<0.01 vs SCH + sulpiride, n = 7, Fig 4Bii, iii, iv). Therefore, phenylephrine alone resulted in a decrease in gamma power whereas together with SCH and sulpiride it resulted in decrease in both theta and gamma power. Overall, these data suggested α1-adrenergic receptor activation was sufficient to decrease oscillatory power, however, they did not provide evidence that DA acts at α1-adrenergic receptors.

**Fig 5:**
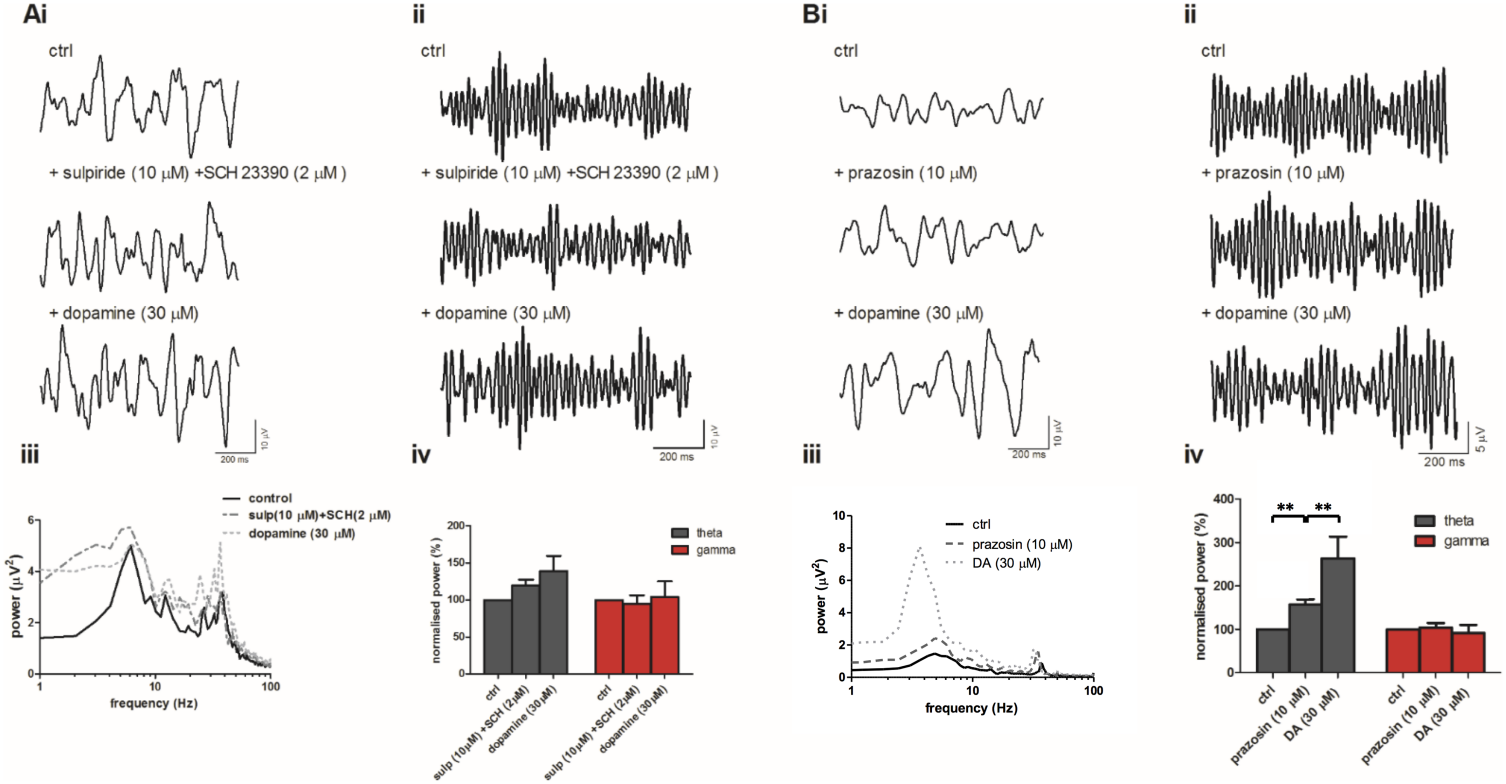
The action of DA is blocked by both DA receptor and α1 adrenergic receptor antagonists. (A) Band-passed raw data of (i) theta and (ii) gamma oscillations after induction with CCh and KA (ctrl) and after application of sulpiride (10 μM) and SCH 23390 (SCH, 2 μM) and then addition of dopamine (30 μM). iii) Typical power spectra demonstrating peak responses before (solid line) and after application of sulpiride and SCH (dashed line) and then dopamine (dotted line). (iv) Peak power changes of theta (grey bars) and gamma (red bars) oscillations normalised to control. (B) Band-passed raw data of (i) theta and (ii) gamma oscillations after induction with CCh and KA (ctrl) and after application of prazosin (10 μM) and then dopamine (30 μM). (iii) Typical power spectra demonstrating peak responses before (solid line) and after application of prazosin (dashed line) and dopamine (dotted line). (iv) Peak power changes of theta (grey bars) and gamma (red bars) oscillations normalised to control **p<0.01.

In order to address this possibility, we applied DA in the presence of DA receptor antagonists and then in the presence of the α1 receptor antagonist prazosin. As predicted, application SCH and sulpiride blocked the DA-mediated inhibition of gamma power (theta; 138.6 ± 21.1% of control, ns, n = 6 5Ai, iii, iv) and (gamma; 104.1 ± 21% of control, ns, n = 6, 5Aii, iii, iv). Application of the α1-adrenergic receptor antagonist prazosin (10 μM) increased theta power (157 ± 12.6%, n = 10, p<0.01, Fig 5Bi, iii, iv) but was without effect on gamma power (103 ± 11.7%, n = 10, ns, Fig 5Bii, iii, iv). Subsequent application of DA further increased theta power (to 263.5 ± 49.8% of control, p<0.01 vs prazosin alone, n = 10, Fig 5Bi, iii, iv) but DA was now without effect on gamma power (91 ± 19.4% of control, n = 10, ns, Fig 5Bii, iii, iv).

## Discussion

In this study we have shown that simultaneous theta (2–8 Hz) and gamma (30–45 Hz) oscillations can be elicited in layer V of M1 *in vitro* and that these oscillations are differentially modulated by DA. Application of DA was without effect on theta oscillatory power but resulted in a significant decrease in gamma oscillatory power. The effect on gamma was mimicked by application of amphetamine and by α1 adrenergic receptor activation but not by selective DA receptor activation. Following antagonism of DA receptors, subsequent α1 adrenoreceptor activation still produced a decrease in gamma power, while following antagonism of α1 adrenergic receptors, DA increased theta power but no increase in gamma power was observed. These results indicate that DA acting at DA and α1 receptors differentially modulates the power of theta and gamma oscillations. The block of DA-mediated effects on gamma power by the α1 adrenergic receptor antagonist prazosin raises the possibility of DA action at α1-adrenergic and/or DA/α1 heteromultimeric receptor complexes.

### Simultaneous theta and gamma oscillations in vitro

Previous research in our laboratory investigated beta oscillations, produced in M1 using 400 nM KA and 50 μM CCh [18]. Since then a modified sucrose-based aCSF has been developed, which incorporated neuroprotectants (to prevent excitotoxicity and cell death) and anti-oxidants (to prevent damage from reactive oxygen species and free radicals), such as indomethacin, uric acid, ascorbic acid, N-acetyl cysteine, taurine and amino-guanidine. Use of this modified aCSF has enabled reduced concentrations of KA (100 nM) and CCh (10 μM) to be used to elicit oscillatory activity at in M1.

Simultaneous oscillations, at theta (6.6 Hz) and gamma (36.6 Hz) which show greatest power in layer V and display significant phase-amplitude coupling have been previously described in our refined *in vitro* preparation [22]. Furthermore, the results of our pharmacological studies are consistent with theta oscillations being generated as a result of synchronous intrinsic membrane potential activity [45,46,47,48,49], while the generation of gamma oscillations require AMPA mediated EPSP/Cs indicating a mechanistic resemblance to the pyramidal-interneuron gamma (PING) model of oscillation generation which depends upon mutually connected excitatory pyramidal cells and inhibitory interneurons [50,51,52]. Using magnetoencephalography in healthy participants we have also previously reported in M1, two similar rhythms at ~10 Hz and 15–30 Hz [19]. The faster rhythm also appears sensitive to GABAA receptor ligands, supporting the view that their generation is comparable between modalities.

### DA receptors and oscillations

With regard to theta activity, application of DA appears to have little effect on oscillatory power. Early studies showed that theta activity was unaffected by the loss of DA [53] and DA had no effect on hippocampal intrinsic membrane potentials which oscillated at theta frequency [54]. Furthermore, *in vivo* studies in the prefrontal cortex and hippocampus indicated that dopaminergic modulation yielded no change in theta power although coherence between the two areas was increased [55], indicating that information sharing between brain regions can be altered regardless of changes in oscillatory power.

In contrast to the lack of action on theta oscillations, bath application of DA produced a moderate but significant decrease in gamma oscillatory power consistent with reports of DA suppressing gamma oscillations in the hippocampus [54,56]. Previously, D1 receptor activation has been shown to increase the excitability of both pyramidal cells and interneurons [57,58], while D2 receptor activation appears to reduce excitability [59]. Similarly, activation of D1-like and D2-like receptors in striatum had opposing effects on GABA release [60]. This led us to speculate that differential effects of applied D1-like and D2-like receptor agonists on oscillatory activity would be observed. However, this was not the case and both D1-like receptor activation with SKF38393 and D2-like receptor activation with quinpirole increased the power of both theta and gamma activity.

The increase in gamma power upon application of SKF was expected as the classical view of D1 receptor activation is to promote network excitability by the direct modulation of voltage-dependent and ligand gated ion channels inducing postsynaptic depolarisation of pyramidal cells and interneurons. D1 receptors positively couple to adenylyl cyclase (AC), elevating levels of cAMP and activating protein kinase A which affects multiple downstream targets which include voltage-dependent Na^+^ channels [57, 61], the activation of L-type Ca^2+^ channels [62,63] and attenuation of a slowly inactivating outward rectifying K^+^ current [57,64].

In contrast, the increased gamma power observed upon application of the D2 agonist quinpirole was unexpected as D2 receptors negatively couple to AC and have been shown to mediate decreases in excitability of PFC layer V pyramidal cells [65]. While it is recognised that decrease in network excitability may lead to changes in firing rate and pattern of firing that could potentially increase network synchrony, enhanced cellular excitability mediated by D2 receptors is not unprecedented. In layer V neurons of PFC, quinpirole has been shown to enhance an afterdepolarisation mediated by L-type calcium channels [66]. Furthermore and specifically in M1 *in vivo*, quinpirole has been shown to increase firing rate [31] while D2 antagonists produce transient reduction in excitability [67]. Further experiments at single-cell level are required to address the possibility that D2-like receptors have an excitatory role in M1.

### α1 receptors and oscillations

Amphetamine was initially applied to promote endogenous release and avoid the problems associated with oxidation of bath-applied DA. However, amphetamine has also been shown to release noradrenaline, 5-HT and acetylcholine as well as DA [40,41,42]. Therefore, we cannot be certain that the effects observed are solely due to action of DA on DA receptors. Indeed, DA action at α1 adrenergic receptors was subsequently confirmed as the selective α1 adrenergic receptor agonist phenylephrine decreased gamma power while the α1 adrenergic receptor antagonist prazosin blocked the effect of DA.

Previous *in vitro* studies have reported a decrease in CCh and KA induced gamma power by noradrenaline in CA3 [56], while specific activation of α1 adrenergic receptors reduced gamma power [68]. In addition, *in vivo*, activation of locus coeruleus (LC) reduced EEG in 20–44 Hz range [69] and a decrease gamma activity in the dentate gyrus [70,71]. Interestingly, while activation of LC reduces gamma it enhances theta frequencies [69]. In M1, this effect was observed upon application of amphetamine but not on activation of α1 adrenergic receptors with phenylephrine.

α1 adrenergic receptor activation may lead to the suppression of excitability through a direct hyperpolarisation of pyramidal cells [68,72,73] and/or a decrease in EPSP amplitude, for example, through presynaptic effects [74]. Similarly, adrenoceptors may mediate a decrease in excitation of interneurons [68,75,76] which would lead to reduced gamma power.

### Promiscuous action of DA and α1 receptors

There is a long literature of promiscuous interaction between DA and adrenergic receptors, particularly α1 [43,77,78]. A recent study by Yang et al., (2014) [79] found that a DA-induced membrane hyperpolarisation was mediated by adrenergic receptors, while DA acting at adrenergic receptors on interneurons of the entorhinal cortex produces membrane depolarisation through the inhibition of an inward rectifier K^+^ channels resulting in secondary activation of T-type calcium channels which augments the frequency of sIPSP and mIPSPs [44].

In prefrontal cortex, D1 receptors and adrenergic receptors appear to co-localise in dendrites of neurons [80] which may suggest interaction between these two receptors at the level of G-protein or second messsenger systems. Furthermore, DA/α1 subunits potentially form heteromultimeric receptor complexes which could display unique signalling and activation properties [81,82,83,84]. The robust effects of DA acting through α1 receptors in M1 is surprising considering DA has only 1/50^th^ the affinity of noradrenaline for the α1 receptor [85]. It may be that DA has a greater affinity for a DA/α1 heteromultimeric receptor than for homomeric α1 receptors.

In summary, we have shown that M1 can generate co-existent theta and gamma oscillations which are differentially modulated by DA acting at DA and alpha-1 receptors.

## Abbreviations

aCSF: artificial cerebral spinal fluid
CCh: carbachol
DA: dopamine
EPSP: excitatory postsynaptic potential
FS: fast-spiking
KA: kainic acid
LFP: local field potential
M1: primary motor cortex
mIPSP: miniature inhibitory post synaptic potential
sIPSP: spontaneous inhibitory post synaptic potential
